# ^1^H-MRS glutamate level predicts auditory sensory gating in alcohol dependence: Preliminary results

**DOI:** 10.1186/s40810-015-0014-8

**Published:** 2015-12-18

**Authors:** Robert J. Thoma, Jason Long, Mollie Monnig, Ronald A. Yeo, Helen Petropoulos, Charles Gasparovic, Jessica Pommy, Paul G. Mullins

**Affiliations:** 1 Departments of Psychiatry and Psychology, University of New Mexico, Albuquerque, NM 87131, USA.; 2 Mind Research Network, Albuquerque, 1100 Yale NE, Albuquerque, NM, USA.; 3 Center for Alcohol and Addiction Studies, Brown University, Box G-S121-5, Providence, RI 02912, USA.; 4 Bangor Imaging Unit, School of Psychology, Bangor University, Adeilad Brigantia, Penrallt Road, Bangor LL57 2ASGwynedd, UK.

**Keywords:** Magnetoencephalography, MEG, Proton magnetic resonance spectroscopy, 1H-MRS, Auditory sensory gating, Paired click paradigm, Auditory event related potential

## Abstract

**Background:**

Impairment in auditory sensory gating (ASG) has been documented in alcohol dependence [1]. Likewise, it has been shown that ASG becomes abnormal during alcohol administration in otherwise healthy individuals [2]. Patterns of gating abnormality associated with alcohol use are likely associated with an alcohol responsive neurochemical like glutamate (Glu), particularly since it is well-established that alcohol affects NMDA receptors and that glutamatergic functioning is abnormal in both acute alcohol use and in alcohol dependence [3]. Hence, a link between Glu metabolite levels and ASG was hypothesized. It was first hypothesized that Glu and ASG abnormality would be found in groups with alcohol dependence. A second hypothesis was that across groups, greater Glu would predict reduced ASG.

**Methods:**

Groups were comprised of healthy, non-drinking controls (Controls, *N* = 4), individuals with current alcohol dependence (AUD-current, *N* = 6), and with alcohol dependence in remission for at least 1 year (AUD-remission, *N* = 6). Participants underwent a diagnostic assessment for alcohol consumption, MRI, 1H-MRS for in vivo assessment of Glu and other metabolites, and MEG scanning during a paired click protocol. ASG was computed as the ratio of the source strength of the 50 ms component in the event related field (ERF) to the second click in the pair divided by the source strength of the 50 ms component to the first click in the pair.

**Results:**

Univariate MANOVAs controlling for age and gender revealed a significant effect for group on Glu and ASG, such that ASG ratios were significantly elevated, implying weakened gating. Glu concentration was reduced in AUD-current relative to the other two groups. Further analysis revealed that when additionally controlling for the group effect, reduced Glu predicted increasing impairment in ASG.

**Conclusions:**

The overall results were consistent with the hypothesis that differences in Glu metabolite levels associated with alcohol dependence result in impaired ASG.

## Background

Auditory sensory gating (ASG) assesses the degree to which redundant auditory information is filtered very early in perceptual processing and is often assessed in terms of the 50 millisecond positivity in the auditory event related potential (AERP; [1, 4, 5]). In a paired click paradigm, pairs of auditory click stimuli are presented with a half-second interstimulus interval. The peak amplitude of the auditory event-related potential (ERP) of the second click in the pair (S2) can be divided by the ERP peak amplitude of the first click (S1) to create a gating ratio—a metric of the extent to which filtering is successful. This model of auditory sensory gating (ASG) has been extensively investigated as an endophenotype for schizophrenia, as sensory gating ratios in this population are reliably higher than in control populations.

Recent research has also indicated that gating abnormalities are associated with alcohol dependence, but much work remains to determine the extent to which gating may be an effect of alcohol use, and if so, what the neural mechanisms are by which gating becomes impaired. Increases in ASG ratios occur with acute alcohol ingestion [2] and the level of ASG impairment in patients with a history of alcohol dependence has been reported to be equal to that seen in schizophrenia [1]. Together, these results suggest that chronic alcohol dependence may result in a lifelong gating impairment. Neurochemical mechanisms underlying ASG have long been of interest in schizophrenia research and a sequence of neurochemical events associated with ASG has recently been described in detail [6]. Excitatory glutamatergic afferents relay auditory sensory information about S1 to hippocampal pyramidal neurons that modulate the response in auditory cortex (ultimately measured as the P50 AERP). A branch of the glutamatergic afferents also excites inhibitory interneurons that serve to reduce subsequent activation of the pyramidal cells. Excitatory cholinergic afferents to these interneurons from the fimbria fornix serve to maintain the gating mechanism, and “conditioning” of auditory sensation can last for as long as several seconds [6].

In alcohol use disorders (AUD), glutamate (Glu) concentrations, measured in vivo using proton magnetic resonance spectroscopy (1H-MRS) in anterior cingulate cortex [3, 7], [8]), frontal white matter [9], and insula [7] are abnormal. In a cross-sectional study, Mon and colleagues [10] showed recovery of Glu levels from 1 to 5 weeks of abstinence in an AUD population. Consistent with Mon et al [10], elapsed time since initiation of successful remission predicted a return of Glu levels to that of controls [3]. Additionally, Glu may play a role in AUD relapse, as levels of both craving and Glu are positively correlated in AUD [11].

Given the clinical importance of understanding alcohol dependence and the associated sensory and behavioral complications that arise, it is worthwhile investigating a possible link between impaired ASG and glutamate levels in alcohol dependence. To do so 1H-MRS neurometabolite concentrations and MEG M50 sensory gating data were collected in groups of healthy controls (Controls), current alcohol dependent (AUD-current) and fully remitted alcohol dependent (AUD-remission) individuals. It was hypothesized that Glu concentration would be abnormal in the AUD groups, and that Glu would account for independent variance in sensory gating ratios across groups. An anterior cingulate cortex (ACC) voxel was selected as the site for 1H-MRS measurement due to the high reliability and validity of neurometabolite measurement in this region [12, 13]. ACC was also selected because M50 modulation is known to have a strong fronto-temporal component [14] and differences in ACC Glu concentration affect fronto-temporal connectivity [15]. Magnetoencephalography (MEG) was used for assessment of gating because MEG temporal resolution is suitable for measurement of the AERF M50, and assessment of ASG in terms of left- and right-hemisphere M50 dipole activity permits investigation of subtle hemispheric differences that may go undetected using other means [5, 16]. In addition, since MEG-assessed signal strength is sensitive to Glu concentration in auditory cortex [17], it was thought to be a particularly good candidate technology for the assessment of sensory gating.

## Methods

### Participants

The present study was approved by the University of New Mexico Health Sciences Center, Human Research Protections Office. Participants were recruited from the general community of the city of Albuquerque, New Mexico, from the University of New Mexico (UNM) Hospital’s Alcohol and Substance Abuse Program (ASAP), and from the UNM Center for Alcoholism, Substance Abuse and Addictions (CASAA). Groups were comprised of healthy controls (Controls, *N* = 4), participants with active alcohol dependence (AUD-current, *N* = 6), and participants with full remission from alcohol dependence for least 1 year (AUD-remission, *N* = 6). Table 1 shows the demographic composition of each group.

General inclusion criteria were (1) age 18–45 years old, (2) ability and willingness to participate in all study components, (3) functional facility with English language, (4) no evidence of fetal alcohol syndrome, (5) no drinking in the 48 h prior to study participation, (6) negative urine sample for presence of illicit drugs, (7) no history of neurological disorder or disease, (8) no history of head injury with loss of consciousness > 5 min, (9) no evidence of psychosis, (10) no current psychoactive medication use, and (11) no diagnosis of mental retardation or of learning disability. General exclusion criteria: 1) DSM-IV diagnosis of cocaine, heroin, methamphetamine, inhalant, or marijuana dependence. Table 2 provides descriptive statistics of the drug and alcohol measures for each group.

Diagnostic determination was made using the Structured Clinical Interview for DSM-IV-IP (SCID-IV; [18]; see also [19]). Inclusion criteria for the AUD-current group (*N* = 6) were (1) diagnosis of alcohol dependence, (2) active alcohol use within the past 30 days (i.e. not in early or full remission), that included at least one episode of binge drinking (5 or more drinks per occasion for males, 4 or more drinks per occasion for females). Inclusion criteria for the AUD-remission group (*N* = 6) were (1) History of alcohol dependence (as determined by the SCID-IV) and (2) at least 1 year of complete remission from alcohol and illicit drug use. The Control group (*N* = 4) was comprised of healthy individuals who were either non-drinkers or mild social drinkers with no history of diagnosis with substance use disorder. General exclusion criteria were (1) diagnosis of schizophrenia spectrum or other psychotic disorder, and (2) a first-degree relative with schizophrenia. Table 3 provides information regarding co-morbid SCIDIP-assessed lifetime diagnoses.

### Procedures

#### MRI and MRS data acquisition

**Fig. 1:**
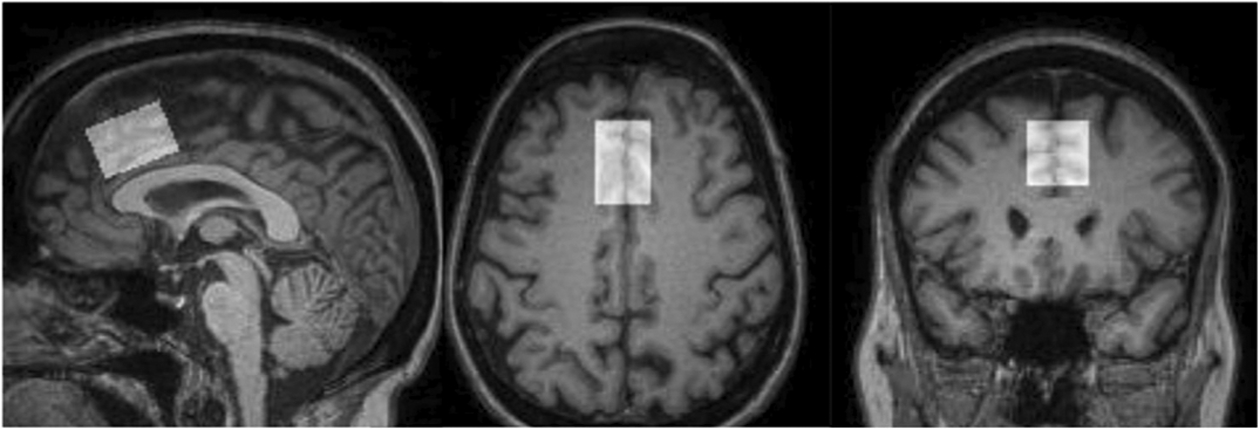
1H-MRS voxel location in medial prefrontal cortex

MRI and 1H-MRS data were collected on a Siemens 3-Tesla TrioTIM scanner using a 12-channel radiofrequency head coil. T1-weighted images were collected in the sagittal plane using a five-echo 3D MPRAGE sequence (TR/TE/TI = 2530/1.64, 3.5, 5.36, 7.22,9.08/1200 ms, flip angle = 7°, field of view = 256 × 256 mm, matrix = 256 × 256, 1 mm thick slice, 192 slices, GRAPPA acceleration factor = 2). Using these images, a single 1H-MRS voxel was positioned in the bilateral medial frontal cortex directly superior to the corpus callosum, containing anterior cingulate, middle frontal, and superior frontal gyri (see Fig. 1; [3]. A PRESS (point-resolved spectroscopy) sequence (TR/TE = 1.5 s/40 ms, voxel size = 20 × 30 × 20 mm, averages = 192) was collected, using an echo time (TE) of 40 ms for improved detection of Glu [12]. An unsuppressed water sequence for use as a concentration reference and eddy current correction in post-processing was collected with 16 averages and otherwise identical parameters for each spectrum. Figure 1 depicts the voxel location in a single MRI.

#### 1H-MRS data analysis

**Fig. 2:**
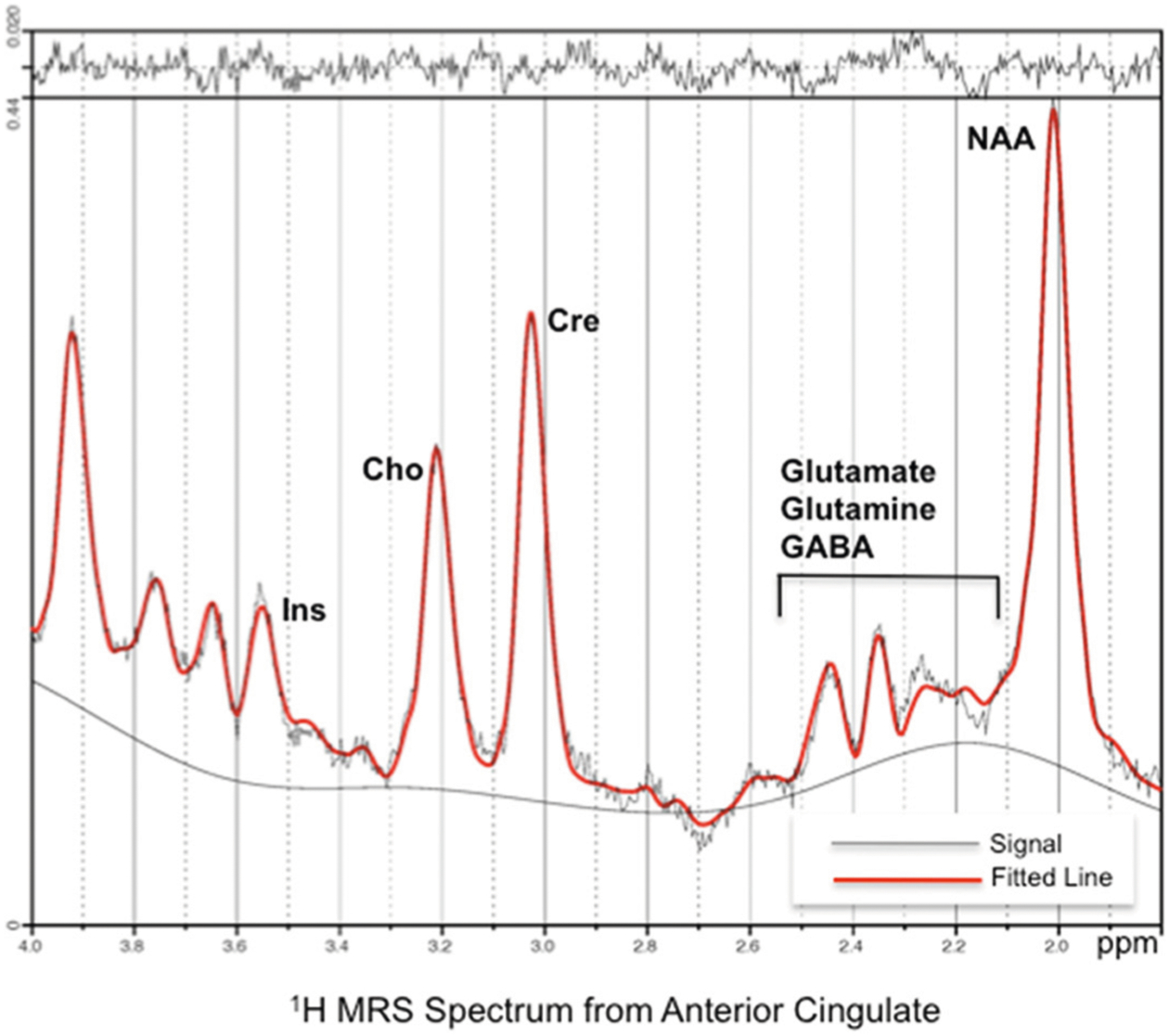
A typical 1H-MRS spectrum for a sample individual

Raw time-domain 1H-MRS data from 4.0 – 1.0 ppm in the spectral dimension were analyzed using LCModel [20] with the unsuppressed water scan as a concentration reference. Parameterized macromolecule intensities were included over the fitted spectral region (the LCModel macromolecule intensity set MM20). As a quality-assurance measure, LCModel produces a Cramer-Rao lower bound (CRLB) of the fit to the peak of interest. If this value was greater than 20 %, the fit was deemed unreliable and excluded from analysis. Metabolite concentrations in molality units of mmol/kg of tissue water were computed for total creatine plus phosphocreatine (Cre), total choline-containing compounds (Cho), myoinositol (Ins), total N-acetylaspartate plus N-acetyl-aspartylglutamate (NAAG), glutamate (Glu), and glutamine (Gln). To calculate brain tissue within the spectroscopic volume, T1-weighted images were segmented into gray matter, white matter, and cerebrospinal fluid (CSF) using SPM5. The spatial coordinates of the voxel and T1-weighted image were used to register the voxel volume to the segmentation maps generated from the T1-weighted image. Gray matter, white matter, and CSF percentage were then determined as the sum of pixels within each tissue type divided by the total pixels in the voxel. Metabolite concentrations were then computed, correcting for partial-volume CSF and T1 and T2 relaxation effects using methods described previously [21]. Fig. 2 shows a representative spectrum from a control participant.

#### MEG auditory paired-click design

Presentation software was fully integrated with CTF data acquisition software to record stimulus presentation in the MEG data file. Three millisecond binaural clicks were presented in pairs, with a 500 millisecond inter-stimulus interval (ISI) between the first (S1) and second (S2) click in the pair. Click pairs were delivered to participants with Etymotic transducers placed outside the shielded room (to control electrical artifact) and conducted through 2.5 meter plastic ear tubes. While there was no detectable jitter in stimulus delivery, sound card and stimulus delivery hardware resulted in a 31 ms stimulus delay that was corrected for during offline processing. Inter-trial interval (latency between click pairs) was randomly varied between 7 and 12 s in 1-second steps. Prior to experimental procedures, a hearing test was performed during which each subject’s hearing threshold was determined using click stimuli presented through the Etymotic sound delivery system. Click stimuli were then presented at 35 dB above this threshold. Click pairs were presented over a roughly 35-minute data recording session.

#### MEG data collection

MEG data were recorded during presentation of the paired-click protocol. The data were recorded on a VSM/CTF-Medtech Omega 275 whole-head biomagnetometer system located at the Mind Research Network Imaging Center (Albuquerque, NM, USA). Vertical and horizontal extra-ocular electrodes [electro-oculogram (EOG)] were placed to record eye movements and two electrodes were placed to record heartbeat. Data were collected with subjects comfortably seated in a soft chair with their head in a Dewar helmet containing the MEG sensors. All data were collected at a digitization rate of 1200 Hz with a 0.1–130 Hz bandpass filter and continuous data were stored for post-processing.

#### Noise reduction

MEG data were recorded within a three-layer (two μ-metal and one aluminum) magnetically shielded room (Vacuumschmelze, GmbH & Co. KG, Hanau, Germany) to reduce interference from ambient environmental magnetic fields to less than 0.15 T. To further reduce environmental magnetic noise, all MEG data were collected applying CTF third-order synthetic gradient computations. To identify eye-blink and muscle artifacts, two channels of electro-oculogram (EOG; vertical and horizontal) were collected concurrently with MEG. Epochs were rejected if peak-to-peak signal strength for the entire epoch exceeded 150 μV in any EOG channel or 3000 fT in any MEG channel. One hundred twenty five click-pairs were presented to each participant and all participants achieved at least 100 artifact-free trials for data averaging. Movement by participants was monitored at all times by research assistants. Additionally, a head localization function within CTF Data Acquisition Software was used to track pre- and post-run head locations. Fiducial coil locations were recorded at the beginning of each data collection run. When recording was complete, a post-run coil localization was implemented allowing computation of how much the head moved (in cm) between the start and end of the recording. Head movement was deemed acceptable if movement values were less than .5 cm in any plane.

#### MEG/MRI coregistration procedures

The purpose of co-registration procedures is to locate the position of the patient’s head on the MRI relative to the MEG channels. This was accomplished by first positioning thee small localization coils at landmark locations (periauricular and nasion fiducial points) on each participant prior to MEG data collection. From these data, the patient’s head was located relative to the coordinate system fixed to the dewar (the CTF dewar coordinate system). A new coordinate frame relative to the patient’s head, known as the CTF MEG head coordinate system was then derived. During coil placement procedures, data for 50 to 100 Polhemus digitizer points were collected to record coil positions and define head shape. Following data collection, CTF MRIConverter software was used to import Magnetic Resonance Imaging (MRI) data and subsequently to produce an isotropic volumetric image file. Within the CTF MRIViewer application, this image file was co-registered with the head coordinate system produced by the CTF MEG System software. Coregistration was then accomplished via a 2-stage process. Initially, the first author (RJT) used MRIViewer interactive software to co-register MEG and MRI data. This co-registration was then reviewed for accuracy by a second research team member.

#### MEG data reduction and source localization

VSM/CTF software was scripted to assure identical data preprocessing across participants. First, to preserve data storage space, data were downsampled to 600 Hz. Second, 4–45 Hz recursive Chebyshev II bandpass filters were applied to the continuous data. Using the CTF interactive software program, MRViewer, a spherical volume conductor approximating the inner skull surface was created. The resultant best-fit sphere was saved in the head model file for dipole localization and in dipole files for source computations. Using the CTF DipoleFit software package, two starting points for the dipole fits were determined, one in each hemisphere. To place these starting points, each individual’s unique spherical head model was first bisected in the plane of the x-axis. Two radii were determined in this plane that connected the center of the sphere to the furthest −y and + y points, and starting points were placed at the midpoints of these radii. The CTF source localization software uses an iterative non-linear minimization algorithm to compute the position and orientation. Data from the full sensor array was used to compute two concurrent, equivalent current dipoles (ECDs), localizing left- and right-hemisphere auditory cortex on posterior, superior temporal gyrus (STG). Only ECDs with goodness-of-fit values exceeding 75 % for S1 were accepted. Once localizations were determined, the M50 peak was defined as the first dorsal-oriented dipole occurring prior to M100 and 40 to 80 ms poststimulus. For the S2 M50 response, it was assumed that the location of the S2 dipole was the same as that of the S1 dipole, and that the latency of the S2 dipole was within 10 ms of that associated with S1. Peak S2 M50 source strength was computed based upon the highest peak within 10 ms of the S1 peak latency. M50 suppression for each hemisphere was expressed as a ratio: S2 dipole peak strength divided by S1 dipole peak strength.

### Statistical analysis

All statistical analyses were done using SPSS-version 20 software. Group differences in 1H-MRS metabolite concentrations were tested using univariate ANOVAs controlling for the effect of age and gender. Group differences in ASG were tested using repeated measures ANOVA, with left- and right-hemisphere ASG ratios included as the repeated measure, group as the between-subjects factor, and age and gender entered as covariates. To assess the independent variance in sensory ASG ratios accounted for by Glu, metabolite values were added to the repeated measures ANOVA as covariates. Since gating ratios were computed on the basis of M50 S1 and S2 peak amplitudes, these variables were considered as within-subjects dependent variables in a repeated measures MANOVA (S1S2 amplitude and Hemisphere were entered as repeated measures), with group as the between subjects factor, and gender and age considered as covariates.

## Results

### 1H-MRS results

Descriptive statistics for 1H-MRS metabolite concentrations are presented in Table 4. Mean Glu level was significantly lower in AUD-current relative to the Control and AUD-remission groups (F(2,9) = 11.83, *p* = .04; partial eta squared = .890). Cramer-Rao lower bounds (CRLB)were computed for all metabolites, and setting a maximum CRLB value of 20 resulted in the rejection of four participant’s Gln values, resulting in a total *N* = 12 for analysis of Gln.

### MEG results

ASG ratios, and M50 amplitude and latencies for S1 and S2 are presented in Table 5. A significant main effect for group was revealed, such that AUD-current had higher gating ratios than the Control and AUD-remission groups (F(2,12) = 6.96, *p* = .01; partial eta squared = .542).

### Comparison of MEG and 1H-MRS

**Fig. 3:**
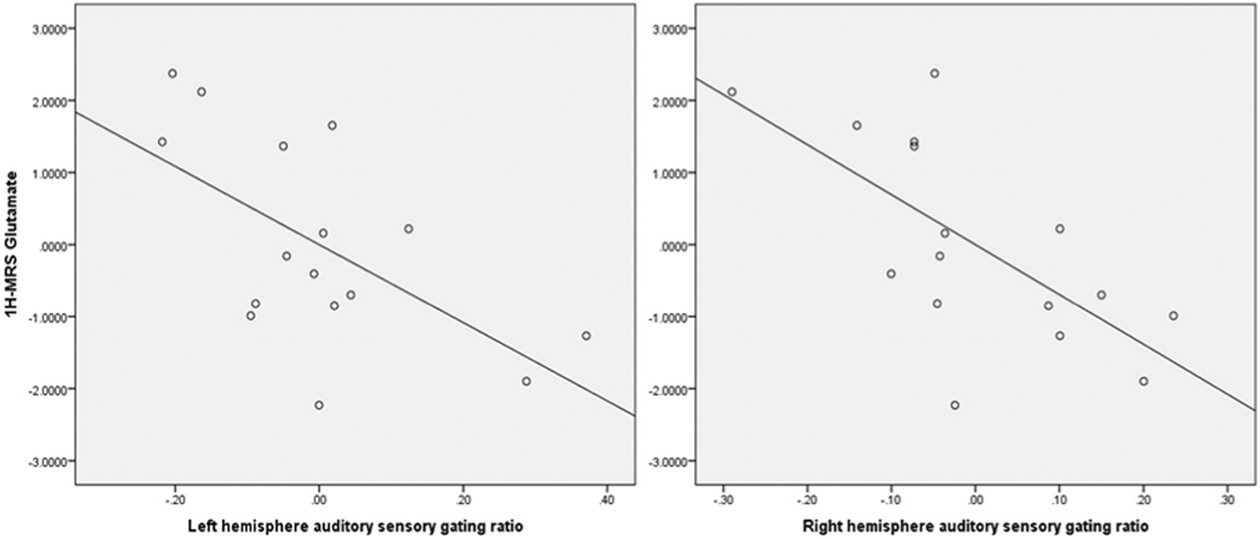
Scatterplots showing distributions of 1H-MRS Glu with left-hemisphere (Beta = −.73, *p* = .001) and right- hemisphere (Beta = −.67, *p* = .004) sensory gating ratios

In analyses using ASG as dependent variables, a significant main effect for Glu was observed (F(2,11) = 11.28, *p* = .006; partial eta squared = .506), such that higher Glu metabolite concentration predicted lower sensory gating ratio bilaterally. Figure 3 shows scatterplots for the relation between Glu and gating ratios. Other neurometabolite values were considered individually as covariates (not corrected for multiple comparisons), and no effects were found for NAA (*p* = .75; partial eta squared = .063), Cr (*p* = .74; partial eta squared = .065), Gln (*p* = .97; partial eta squared = .006), Cho (*p* = .49; partial eta squared = .044) or Ins (*p* = .43; partial eta squared = .171).

In analyses using S1S2 amplitude as dependent variables (S1S2 amplitude and Hemisphere were entered as repeated measures), a significant main effect was found for Group (F(2,11) = 7.17, *p* = .03; partial eta squared = .443), such that the current-AUD group had higher ECD amplitudes overall. No main effect was found for Glu (*p* = .89; partial eta squared = .002), but a significant two-way Glu*S1S2 amplitude interaction emerged (F(1,9) = 5.80, *p* = .04; partial eta squared = .345). To test the independent effect of Glu on S1 and S2 amplitude, a regression analysis was used, with Glu as the dependent variable and mean S1 and S2 (averaged across hemisphere) and age as predictors. Age (beta = .63, *p* = .01) and S1 amplitude (beta = .65, *p* = .03) were positively correlated with Glu level, and S2 amplitude was negatively correlated with Glu level (beta = −.95, *p* = .005).

In analyses using S1S2 latency as dependent variables, similar repeated measures MANOVA were used (S1S2 latency and Hemisphere were entered as repeated measures), with Group as the between-subjects factor, and Gender and Age considered as covariates. A main effect of Group was found (F(1,9) = 4.35, *p* = .05; partial eta squared = .491), such that the AUD-current group had a shorter mean latency relative to the Control and AUD-remission groups. No effect was found for Glu (*p* = .59; partial eta squared = .045) when it was added as a covariate.

## Discussion

The primary hypothesis that impairment in ASG would be found in the AUD-current and AUD-remission groups was only partly supported. ASG ratios were found to be equivalent in the Control and AUD-Remission groups, and significantly elevated only in the AUD-current group. A test of the second hypothesis, that Glu level would independently predict ASG ratios was supported, in that greater Glu concentration strongly predicted ASG ratios whether the effects of group, age and gender were controlled for or not. To better understand the nature of this effect, gating, latency and amplitude of the subcomponent measure comprising the ASG ratios were analyzed with respect to Glu. Averaging across hemispheres, Glu was positively correlated with S1 amplitude, negatively correlated with S2 amplitude, and unrelated to latency.

Although not entirely consistent with the initial hypotheses, the pattern of results indicates important relationships between AERPs, auditory processing, and Glu levels. Consistent with Glu’s role as the brain’s primary excitatory neurotransmitter, elevated Glu levels predicted greater S1 amplitude. The finding that increased Glu levels are related to *reduced* S2 amplitude are consistent with findings of induced impairment in mismatch negativity (MMN) and P3 oddball (P3) paradigms with ketamine challenge. Ketamine is a Glu antagonist, and ketamine induced changes in AERP-related modulation assessed using MMN and P3 involve the reduction of the typical MMN and P3 effect size [22]. Consistent with these findings, improvement in auditory processing, assessed in terms of reduced ASG ratio, was predicted by higher Glu levels across groups. The results of this study are also consistent with the findings of Chang et al. [23], showing that higher Glu level was associated with smaller S2 amplitude in ASG gating.

The present findings are also generally consistent with the prevailing literature regarding disruption of Glu system functioning in AUD [3]. That Glu concentrations assessed in ACC were associated with the sensory gating system, which is purportedly mediated by medial temporal cortex functioning [6], was not surprising, as Glu is ubiquitous in the cerebral hemispheres and is the major excitatory neurotransmitter in the brain. It is only possible to speculate about the mechanism underlying the relationship between Glu and sensory gating based upon present data, though the current hypotheses were conceived on the basis of our knowledge of Glu changes in alcohol dependence. Alcohol binds to the N-methyl-D-aspartate Glu receptor (NMDAR), ultimately enhancing NMDAR gene expression and changing patterns of membrane depolarization [24]. Chronic alcohol ingestion inhibits the NMDAR functioning and can result in neuronal hyperexcitability and excitotoxicity upon withdrawl [25–29].

## Conclusions

The results of this study are complicated by many factors, perhaps the most serious of which is the small sample size. To address that issue, effect size statistics were included in the reported results. The group effect on neuromtabolites Gln, Glx, NAA, and Ins were of moderate effect size, emphasizing the importance of replication in a larger sample. Similarly, there is evidence of a lateralization effect with regard to S1 signal strength in the MEG data for the AUD-current group, further investigation of which necessarily warrants a larger study. Measurement of neurometabolite levels in event-related dipole locations might have added greater face validity to the present Glu findings. In addition, accurate measurement of GABA, which requires special acquisition procedures owing to overlapping resonances with Cre, Glu and Gln, would help elucidate the overall glutamatergic turnover in these subjects. and perhaps shed light on the role of Gln, which in this study was found to be elevated in both AUD-current and AUD-remission, and which is consistent with previously published results [3].

Both Glu metabolite levels [30] and auditory sensory gating ratio [6] are known to be abnormal in schizophrenia. As the effect of comorbidity of alcohol use disorders and schizophrenia on ASG is additive [3], one potentially important next step is to investigate how Glu and other neurometabolite metabolite levels are affected by the use of alcohol in schizophrenia. In the present study, sensory gating was defined only in terms of the 50 ms component of the event related field, partly to constrain the number of hypotheses tested in this small, but clinically interesting sample. It is known, however, that gating ratios based upon other components of the auditory evoked potential in a paired-click paradigm (e.g., N100, P200) contain additional and independent information [31], and a next step in this research might be to test for relationships between neurometabolite levels and ASG computed in terms of possible neurophysiologic components.

## Figures and Tables

**Table 1:** Group demographics

	Controls	AUD-Current	AUD-Remission
Age (in years)	36.50 (10.91)	37.50 (7.87)	34 (4.69)
Gender	1 M/3 F	4 M/2 F	5 M/1 F
Education (in years)	14.25 (1.71)	13 (1.79)	13 (1.79)

**Table 2:** Alcohol and other substance use variables by Group

	Controls	AUD-Current	AUD-Remission
DrInC total score	1.75 (2.06)	40.67 (4.23)	0
Form-90 Drinks Per Drinking Day (DPDD)	1.34 (1.86)	19.19 (6.94)	0
Form-90 Percent Days Drinking (PDD)	3.28 % (5.65 %)	29.02 % (18.59 %)	0 % (0 %)
Form-90 lifetime weeks nicotine use	0 (0)	521.33 (546.84)	585.00 (459.77)
Form-90 number of days nicotine use	0 (0)	63.17 (48.99)	67.50 (37.65)
Form-90 number of cigarettes per day	0 (0)	11.33 (12.36)	5.00 (4.56)
Cannabis (lifetime use)	0 yes	4 yes	4 yes
Stimulants (lifetime use)	0 yes	2 yes	3 yes
Opioid (non-prescription; lifetime use)	0 yes	1 yes	1 yes
Cocaine (lifetime use)	0 yes	4 yes	4 yes
Hallucinogens (lifetime use)	0 yes	1 yes	2 yes

**Table 3:** Lifetime DSM-IV diagnoses by Group (number of subjects reporting history of diagnosis)

	Controls	AUD-Current	AUD-Remission
Bipolar disorder	0	1	0
Depression	0	3	4
Dysthymia	0	2	1
Brief psychotic episode	0	0	1
Panic disorder	0	3	0
Agoraphobia	0	0	1
Social phobia	0	0	1
Obsessive compulsive symptoms	0	0	1
Posttraumatic stress disorder	0	2	1
Generalized anxiety disorder/anx dis NOS	0	6	1

**Table 4:** Neurometabolite levels by Group

	Control	AUD-current	AUD-remission	*p*-value^a^	Partial eta squared
Glu	13.77 (1.17)	12.53 (1.27)	12.99 (1.74)	.04	.89
Gln^b^	3.59 (0.49)	6.83 (2.36)	5.88 (2.72)	.62	.68
Glx	17.03 (1.87)	18.78 (3.56)	18.34 (3.10)	.14	.80
NAA	15.84 (0.36)	15.80 (1.25)	15.39 (0.72)	.36	.61
Cre	13.42 (0.83)	13.61 (1.29)	14.16 (0.87)	.87	.13
Ins	11.82 (1.61)	13.25 (2.02)	13.39 (0.65)	.27	.71
Cho	2.89 (0.17)	3.01 (0.32)	3.29 (0.36)	.90	.09

a Overall *p*-value for the effect of group, controlling for age and gender

b Based upon *N* = 12 (1.7)

**Table 5:** M50 S1 and S2 amplitude and latency values by Group [mean (standard deviation)]

	Control		AUD-current		AUD-remission	
	M50 peak amplitude (nAm)	M50 peak latency (ms)	M50 peak amplitude (nAm)	M50 peak latency (ms)	M50 peak amplitude (nAm)	M50 peak latency (ms)
Left hemisphere S1	13.95 (4.45)	64.83 (15.36)	22.76 (6.13)	54.45 (9.51)	15.34 (5.13)	66.66 (6.96)
Left hemisphere S2	6.34 (1.99)	62.25 (15.52)	15.83 (5.32)	52.86 (6.92)	7.98 (2.90)	65.34 (3.62)
Left hemisphere (S2/S1)	.47 (.09)	NA	.69 (.18)	NA	0.52 (.10)	NA
Right hemisphere S1	12.42 (3.36)	62.78 (10.79)	10.72 (.87)	50.57 (8.06)	11.90 (2.45)	63.00 (8.98)
Right hemisphere S2	6.25 (2.02)	62.75 (13.22)	6.77 (1.01)	55.27(7.76)	4.95 (1.75)	60.68 (9.22)
Right hemisphere S2/S1	.44 (.02)	NA	.58 (.13)	NA	.40 (.14)	NA
